# Physician-Assisted Dying Witnessed by Emergency Medical Services: A Case Report

**DOI:** 10.5811/cpcem.38060

**Published:** 2025-03-20

**Authors:** Saahith Potluri, Tharun Potluri, Jose Victor Nable, Paul Peng, Kusum Punjabi

**Affiliations:** * Krieger School of Arts and Sciences, Johns Hopkins University, Baltimore, Maryland; † Montgomery Emergency Medical Services, Belle Mead, New Jersey; ‡ Georgetown University, Department of Human Science, School of Health, Washington, DC; § Georgetown University, Georgetown Emergency Response Medical Service, Washington, DC; ¶ Georgetown University School of Medicine, Department of Emergency Medicine, Washington, DC; || Rutgers Robert Wood Johnson Medical School, Department of Emergency Medicine, New Brunswick, New Jersey

**Keywords:** physician-assisted dying, emergency medical services, refusal of care, protocols, case report

## Abstract

**Introduction:**

Physician-assisted dying (PAD) is a practice that allows terminally ill patients to self-administer prescribed lethal medication. In the 11 states in the United States where PAD is legal, the incidence of PAD cases is rapidly rising. Despite most of these cases occurring in the out-of-hospital setting, states lack specific emergency medical services (EMS) protocols to guide prehospital responders who may encounter PAD in the field. We report a case in which a patient called 9-1-1 for a medical emergency and requested to ingest her prescribed lethal medication while in EMS care.

**Case Presentation:**

Emergency medical services was dispatched for a 56-year-old female bleeding from her tracheostomy stoma. Despite the EMS responders’ recommendation, the patient refused transport and instead requested to ingest her PAD medication. The crew, unfamiliar with PAD laws, were unsure whether they could legally honor the patient’s refusal. Clinicians consulted with online medical control, who were also unaware of PAD. After extensive deliberation, the crew decided to honor the patient’s refusal and thoroughly document the situation. The patient self-administered her medication as EMS cleared the scene.

**Conclusion:**

This case highlights logistical challenges and ethical dilemmas faced by EMS responders and underscores the complexity of balancing patient autonomy with legal and medical responsibilities in prehospital situations involving PAD. As PAD becomes increasingly prevalent, equipping EMS responders with clear protocols and providing ongoing education about prehospital PAD cases are vital for preserving patient rights while protecting the responders from legal and ethical uncertainty.

## INTRODUCTION

Physician-assisted dying (PAD)—also referred to as medical aid in dying (MAiD), physician-assisted suicide, and death with dignity—is a medical practice involving the prescription of lethal medication to a requesting patient who intends to end their life.^1^ It is important to distinguish PAD from euthanasia, which is illegal in the United States. Euthanasia refers to a physician directly administering lethal medication to a patient, whereas PAD refers to a prescription of lethal medication to be voluntarily self-administered by a patient.^2^ Since the legalization of PAD in Oregon in 1997, the practice has become increasingly sanctioned across the US.^3^ Currently, 20% of US residents live in the 11 states where PAD services are legal, and an additional 12.5% live in the five states where PAD is on the legislative agenda.^4^ Since its initial legalization in 1997, the incidence of PAD cases across participating states has rapidly increased each year.^5^

While existing PAD literature largely focuses on PAD in the emergency department setting, a vast majority of PAD deaths occur in out-of-hospital settings, such as the patient’s home or other hospice and comfort-care environments.^5^ In cases where the dying process is interrupted by incomplete ingestion of medication, unanticipated medical emergencies, or other variables, emergency medical services (EMS) may be called to assist. Currently, these cases are seemingly few. In Oregon, which has the most longitudinal and comprehensive PAD data to date, EMS is involved in fewer than 1% of PAD deaths every year.^6^ Consequently, there is a paucity of literature surrounding the involvement of EMS responders in prehospital PAD situations. Although EMS involvement may be rare, as the incidence of PAD cases rises the frequency of EMS encounters with PAD will likely increase.

Situations involving the self-administration of lethal medication can occur in diverse settings and in circumstances that can complicate EMS responsibilities on scene. To guide first responders in PAD situations, which often require quick decision-making with limited information and heightened emotions from patients and bystanders, clear protocols are imperative. As is described in the following case report, prehospital PAD cases pose unique ethical and legal challenges to EMS responders that should be considered and integrated into EMS protocols and training.

## CASE REPORT

A Basic Life Support and Advanced Life Support crew was dispatched with township police to a residence for the report of a 56-year-old female hemorrhaging from a surgical site. Upon EMS arrival, the patient was found lying supine on a bathroom floor. Family members present on scene reported they were changing the dressings around the patient’s tracheostomy stoma when the site began to bleed. Bleeding was controlled using gauze prior to EMS arrival; the first responders estimated 500 milliliters of blood loss. The crew ensured a patent airway and completed an initial assessment. The patient was fully alert and oriented. She had a history of stage IV laryngeal cancer and communicated nonverbally using a notepad. Initial vital signs were pulse rate of 125 beats per minute, respiratory rate of 16 breaths per minute, and an oxygen saturation of 94%. A blood pressure reading could not be obtained due to limited access to the patient’s upper extremities. Given the patient’s blood loss and potential for further hemorrhage, urgent transport to the emergency department was recommended. However, the patient indicated that she did not want to be transported and wrote on the notepad: “I want MAiD now.” The family members presented the crew with MAiD paperwork, hospice care paperwork, and a Physician Orders for Life-Sustaining Treatment (POLST) form that indicated the patient had a do-not-resuscitate (DNR) order. While familiar with hospice care and POLST, none of the EMS professionals or law enforcement officers had previously heard of MAiD.

**CPC-EM Capsule**

What do we already know about this clinical entity?

*Physician-assisted dying (PAD), a legal medical practice in 11 states, enables terminally ill patients to end their lives with prescribed lethal medication*.

What makes this presentation of disease reportable?

*Patients enrolled in PAD programs have official documentation verifying their enrollment and may have do-not-resuscitate orders and/or hospice paperwork*.

What is the major learning point?

*To protect emergency medical service responders from legal and ethical uncertainty, agencies must equip them with clear protocols and education about PAD practices*.

How might this improve emergency medicine practice?

*By knowing how to navigate PAD in the field, first responders can better manage complex scenarios, including refusal of care and complications such as failed ingestion*.

Although the patient was alert and oriented, and understood the severity of her illness, the EMS crew questioned whether they could complete a refusal of care if the patient intended to end her life. Despite the crew’s recommendation, she remained firm in her decision to not want to be transported and to ingest her medication instead. On-line medical control (OLMC) was contacted for guidance. The OLMC physician indicated that they were also unaware of MAiD but directed the on-scene EMS crew to continue following their refusal protocols. During this time, the family members were actively preparing the patient’s MAiD medication while the EMS responders continued to be uncertain about whether it was appropriate for the patient to proceed with medication ingestion.

Law enforcement officers, who were concerned about foul play and potential legal wrongdoing, requested that their supervisor respond to the scene and ensured that their body cameras were recording. Seeking further clarity, EMS requested to speak via telephone to a representative from the medical practice that had prescribed the patient’s lethal medication. The representative, an advanced practice provider, explained the legal basis for MAiD and clarified that the patient was able to ingest the medication at home at any time of her choosing. After nearly an hour of discussion with supervisors, the representative from the prescribing medical practice, and family members, the EMS crew decided to document the patient’s refusal of care and obtain witness signatures from law enforcement and the family. Then the family members, holding a pharmacy bag of compounded medication and a glass of water with sugar, entered the bathroom and closed the door. The EMS crew cleared the scene and returned to service while law enforcement remained on scene to complete their report and await the arrival of a hospice nurse who could pronounce the patient deceased.

## DISCUSSION

This case illustrates the complexities of PAD situations in the setting of prehospital emergency care. Despite familiarity with hospice and POLST protocols, the EMS crew’s unfamiliarity with PAD led to significant confusion on how to proceed. Given the knowledge that the patient intended to end her life, a key challenge for the crew was in deciding whether to allow the patient to ingest her PAD medication while in their care or document the refusal of care and clear the scene prior to medication ingestion. Another challenge was in deciding whether the patient even met criteria for refusal of care. The EMS crew was uncertain whether the patient’s plan for PAD could be interpreted as suicidal ideation, which would disqualify her from refusing care.

Medical psychologists and proponents of PAD argue that suicide and PAD are distinct.^7^–^9^ While suicide is associated with individuals experiencing psychological suffering or emotional distress who wish to end their lives, PAD specifically involves terminally ill patients who, after thorough consultation and contemplation, make a medically sanctioned decision to end their lives. This distinction implies that intending to end one’s life by means of PAD may not necessarily prevent a patient from refusing care. First responders of all levels receive foundational education on ethical principles, including patient autonomy, informed consent, and handling of advanced directives. However, in complex or unclear situations, as with PAD, EMS first responders should not be left to navigate decisions without clear protocol guidance.

In situations where the decision-making process is protracted by an absence of defined protocols, clear communication between all parties and involvement of additional resources is paramount. Delays in responders’ actions stemming from uncertainty on how to proceed can provoke frustration from the patient and their family, who may perceive them as obstacles to the patient’s final wishes. Additionally, EMS responders should acknowledge gaps in their familiarity with PAD and be prepared to seek additional guidance from supervising staff or OLMC. In this case, where supervisors and OLMC were also unfamiliar with PAD, additional guidance was sought by contacting the prescribing medical practice. This step clarified the legal basis for the patient’s request, helping the responders navigate the situation.

In contrast to this case, there could be instances in which EMS is requested for complications that arise from a patient’s ingestion of PAD medication. Commonly reported complications include difficulty swallowing, regurgitation, seizures, and regaining consciousness after ingestion.^10^,^11^ Additionally, delays in death due to prolonged time to effect of lethal medication can lead to patient and family distress, prompting them to contact EMS. In the US, the current medication regimen for PAD commonly includes a combination of digoxin, diazepam, morphine, amitriptyline and, in some cases, phenobarbital.^11^,^12^ Variations in medications, dosages, and individual patient metabolism may contribute to the wide variability in the time from ingestion to death, which can range from minutes to over 100 hours.^6^,^10^ All EMS responders who might encounter PAD patients should be familiar with the medications commonly prescribed, their mechanism of action, and the wide variability in their time to effect.

The ingestion of lethal medication not only introduces medical complexities but also raises situational challenges. Responders may have difficulty differentiating a patient’s intent of PAD from suicide, particularly if the patient ingested their lethal medication alone or without informing others. This can be further complicated when PAD documentation, often unfamiliar to EMS, is present, but no DNR or POLST forms are available. Given a lack of clear documentation, EMS responders may be forced to resuscitate the patient against their wishes. In PAD-participating states, there is minimal regulation surrounding the ingestion of PAD medication at home: healthcare clinicians do not need to be present, and there is no requirement either for informing family members or for an accompanying DNR order.^13^ Thus, EMS responders need to be well-informed and properly equipped with standardized protocols to navigate uncertain situations and ensure consistency in care.

None of the 11 states where PAD is currently legalized include provisions in their legislation regarding the possibility that a PAD patient may require care from EMS. While one state, Maine, mentions PAD in its EMS protocols, none provide specific guidelines for EMS management of such cases, and few discuss the management of patients with terminal illness (Table).

With the rising incidence of PAD nationwide, the lack of PAD-specific guidance in state EMS protocols highlights a significant gap in emergency response preparedness. Emergency medical services responders should be able to rely upon guidelines that outline procedures for proper identification of PAD intent, refusal of care, withholding of resuscitation, and continuity of care. Accordingly, we present a sample EMS protocol that outlines basic procedures for clinicians when responding to a call involving PAD (Figure). While this protocol was independently developed, it expands upon a sample EMS protocol published in 2007 in the Oregon Death with Dignity Act Guidebook for Health Care Professionals.^14^ We encourage state EMS programs and regulatory bodies to consider adopting similar PAD protocols within their practice guidelines.

## CONCLUSION

This case report outlines relevant logistical challenges and ethical considerations for prehospital professionals who may encounter PAD situations. As more states adopt PAD legislation, millions more individuals will be able to obtain prescriptions for lethal medication. It is crucial for state regulatory bodies to ensure that emergency responders, including EMS professionals, medical control, and law enforcement, are equipped with the knowledge, protocols, and ongoing education to appropriately handle PAD situations. Moreover, PAD patients and their families should be provided with resources to help them understand what to expect if they request an EMS response. By working toward these provisions, states can foster an emergency medical system that is better prepared for the challenges of PAD in the prehospital environment.[Fig f1-cpcem-9-182]

## Figures and Tables

**Figure f1-cpcem-9-182:**
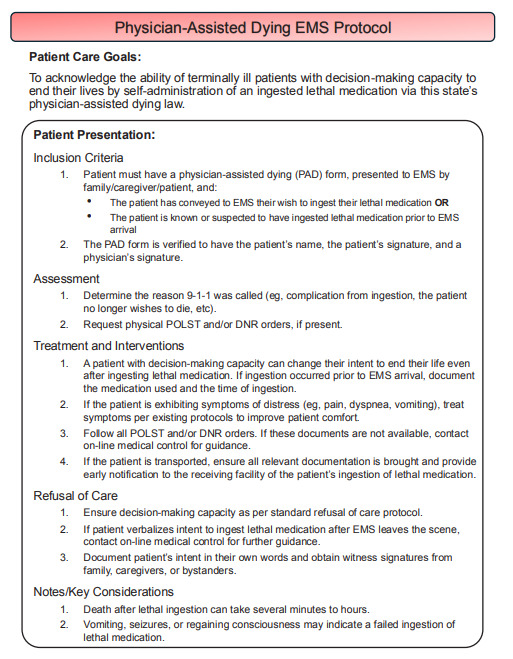
A sample emergency medical services protocol to guide the prehospital management of patients participating in physician-assisted dying. *EMS*, emergency medical services; *POLST*, physician orders for life-sustaining treatment; *DNR*, do not resuscitate; *ED*, emergency

**Table:** Physician-assisted dying (PAD) legislation mentioning emergency medical services (EMS), and EMS protocols mentioning PAD or terminal illness in the 11 PAD-legalized states. Data was collected directly from PAD legislative texts and published statewide EMS protocols. States without statewide EMS protocols are denoted with N/A.

Jurisdiction	Authorization Year	Legislation	EMS mentioned in PAD Law?	PAD mentioned in EMS protocol?	Terminal illness mentioned in EMS protocol?
Oregon	1997	Death with Dignity Act	No	N/A	N/A
Washington	2008	Death with Dignity Act	No	No	Yes
Montana	2009	Rights of the Terminally Ill Act	No	No	No
Vermont	2013	Patient Choice and Control at the End-of-Life Act	No	No	No
California	2015 (reauthorized in 2021)	End-of-Life Options Act	No	N/A	N/A
Colorado	2016	End-of-Life Options Act	No	N/A	N/A
District of Columbia	2017	Death with Dignity Act	No	No	Yes
Hawaii	2018	Our Care, Our Choice Act	No	No	No
New Jersey	2019	Medical Aid in Dying for the Terminally Ill Act	No	No	No
Maine	2019	Death with Dignity Act	No	Yes	No
New Mexico	2021	Elizabeth Whitefield End-of-Life Options Act	No	No	No

*EMS*, emergency medical services; *PAD*, patient-assisted dying; *N/A*, not applicable.
